# Variation in faecal testosterone levels in male gray whales on a foraging ground relative to maturity and timing

**DOI:** 10.1093/conphys/coae094

**Published:** 2025-01-20

**Authors:** A Fernandez Ajó, C L Buck, K E Hunt, E Pirotta, L New, D Dillon, K C Bierlich, L Hildebrand, C N Bird, L G Torres

**Affiliations:** Geospatial Ecology of Marine Megafauna Lab, Marine Mammal Institute, Department of Fisheries, Wildlife and Conservation Sciences, Oregon State University, 2030 SE Marine Science Dr, Newport, OR 97365, USA; Department of Biological Sciences, Northern Arizona University, 617 S. Beaver St., Flagstaff, AZ 86011, USA; Smithsonian-Mason School of Conservation & Department of Biology, George Mason University, 1500 Remount Rd, Front Royal, VA 22630, USA; Centre for Research into Ecological and Environmental Modelling, University of St Andrews, Buchanan Gardens, St Andrews, KY16 9LZ, UK; Department of Mathematics, Computer Science and Statistics, Ursinus College, 601 E Main St, Collegeville, PA 19426, USA; Department of Biological Sciences, Northern Arizona University, 617 S. Beaver St., Flagstaff, AZ 86011, USA; Wildlife and Ocean Health Program Anderson Cabot Center for Ocean LifeNew England Aquarium, New England Aquarium, 1 Central Wharf, Boston, MA 02110, USA; Geospatial Ecology of Marine Megafauna Lab, Marine Mammal Institute, Department of Fisheries, Wildlife and Conservation Sciences, Oregon State University, 2030 SE Marine Science Dr, Newport, OR 97365, USA; Geospatial Ecology of Marine Megafauna Lab, Marine Mammal Institute, Department of Fisheries, Wildlife and Conservation Sciences, Oregon State University, 2030 SE Marine Science Dr, Newport, OR 97365, USA; Geospatial Ecology of Marine Megafauna Lab, Marine Mammal Institute, Department of Fisheries, Wildlife and Conservation Sciences, Oregon State University, 2030 SE Marine Science Dr, Newport, OR 97365, USA; Geospatial Ecology of Marine Megafauna Lab, Marine Mammal Institute, Department of Fisheries, Wildlife and Conservation Sciences, Oregon State University, 2030 SE Marine Science Dr, Newport, OR 97365, USA

**Keywords:** Enzyme immunoassay, Gray whale, Male reproduction, Pcfg, Testosterone

## Abstract

Understanding wildlife reproductive seasonality is crucial for effective management and long-term monitoring of species. This study investigates the seasonal variability of testosterone in male Pacific Coast Feeding Group (PCFG) gray whales, using an eight-year dataset (2016–2023) of individual sightings, drone-based photogrammetry and endocrine analysis of faecal samples. We analyzed the relationship between faecal testosterone levels and total body length (TL), body condition (body area index, BAI), sexual maturity and day of the year using generalized additive mixed models. Our findings reveal a significant increase in faecal testosterone levels in mature males (MM) towards the end of the foraging season. This increase was not observed in JM, highlighting age-dependent development of sexual characteristics. No significant relationship was found between testosterone levels and TL. Additionally, BAI was not significantly associated with testosterone levels. Our results suggest that the increasing testosterone levels in MM gray whales may indicate preparation for mating before the southbound migration. These findings provide valuable insights into the reproductive biology of PCFG gray whales and underscore the importance of non-invasive faecal sampling for studying reproductive seasonality in large whales. Our approach not only provides further insights into the seasonality of male reproduction for the PCFG gray whales but also offers tools to enhance the understanding of male reproduction in baleen whales broadly with non-invasive approaches.

## Introduction

Understanding the spatiotemporal patterns of wildlife reproductive cycles provides critical information for the development of effective management strategies to mitigate human disturbance on reproductive performance. Furthermore, discerning reproductive trends of a population is an essential component of long-term monitoring of any species, as significant deviations from the reproductive patterns of a healthy, growing population may indicate broader changes in the ecosystem (Eberhardt, 2002). Such reproductive information is particularly relevant for small populations with limited ranges, given their increased vulnerability to environmental changes and human disturbance (Willi *et al.*, 2006).

Reproduction in seasonally breeding mammals is often characterized by annual cycles in the reproductive hormones, which are triggered by changes in photoperiod or other environmental cues, as well as by endogenous circannual cycles (Norris and Lopez, 2011). Testosterone, a steroid hormone secreted from the testes, is one of the main androgens in mammals. Elevated androgens are necessary to support testicular maturation and spermatogenesis, and influence expression of reproductive behaviours such as courtship, mating, male–male competition (Buck and Barnes, 2003; Atkinson and Yoshioka, 2007) and circannual timing (Richter *et al.*, 2017). In seasonally breeding vertebrates, testosterone concentrations measured in plasma and faeces typically begin increasing approximately 1–3 months before the breeding season, as testicular recrudescence and spermatogenesis require several weeks of preparation before functional sperm can be produced (Bronson, 1989). Testosterone then typically reaches an annual peak during or just before the breeding season. The occurrence and amplitude of this testosterone peak can vary with age, with immature males (juvenile males, JM) often exhibiting low testosterone concentrations even during the breeding season. Once sexual maturity is reached, mature males (MM), typically present dramatic increases in testosterone concentrations during the breeding season (Beehner *et al.*, 2009). In some species, the amplitude of seasonal testosterone peaks can decline in older males, likely due to reproductive senescence (Hunt *et al.*, 2022). Testosterone concentrations can also vary relative to body size and body condition of an individual, which can impact the physiology, behaviour, timing of sexual maturity and reproductive attempts of mammals (Buck and Barnes, 1999; Hau *et al.*, 2017; Williams *et al.*, 2017). This variability due to age and body condition, along with additional factors such as social cues, exposure to stressors and past experiences, contributes to strong individual differences in testosterone patterns (Sapolsky and Wingfield, 2003; Romero and Wingfield, 2016; Hunt *et al.*, 2018).

Testosterone, like other steroid hormones (e.g. progestins, estrogens, androgens, glucocorticoids, mineralocorticoids), plays a key role in reproduction and stress responses in mammals. As a result, hormone quantification is widely used as a biomarker to monitor stress and reproductive status in wildlife, including cetaceans (Goymann, 2012; Madliger *et al.*, 2018). Steroids are primarily cleared from the bloodstream by the liver, excreted into the gut via bile ducts and modified by gut microbiota, producing 'faecal hormone metabolites' that are excreted in faeces, with some also excreted in urine (Palme *et al.*, 1996, 2005; Goymann, 2012). Faecal hormone metabolites can be measured using antibodies that bind to the parent hormone and ideally show high cross-reactivity with common mammalian faecal metabolites (Schwarzenberger *et al.*, 1996; Palme *et al.*, 2005). The time between hormone secretion and its excretion in faeces depends on species-specific clearance rates and intestinal transit time, typically ranging from 1 to 2 days in large mammals (Palme *et al.*, 1996; Wasser *et al.*, 2000), so hormone assessments from faecal samples provide an integrated measurement of the endocrine state of the individual during that time (Lemos *et al.*, 2020). Decades of validation studies in terrestrial and marine vertebrates confirm that faecal hormone metabolite analysis is a reliable, non-invasive method for assessing various steroid hormones (Wasser *et al.*, 2010; Hunt *et al.*, 2013; Madliger *et al.*, 2018; Palme, 2019).

Seasonal testosterone patterns are well-documented in many male vertebrates, including terrestrial mammals, pinnipeds, and odontocetes (Kellar *et al.*, 2009; O’Brien *et al.*, 2017; Richard *et al.*, 2017; Funasaka *et al.*, 2018; Husak *et al.*, 2021). Less is known, however, about testosterone patterns in mysticetes (baleen whales). Baleen whales typically undergo annual migrations from high-latitude feeding grounds in summer to subtropical breeding grounds in winter. Calving generally occurs during specific portions of the winter months (Lockyer, 1981), which suggests a regular alternation between reproductively active and inactive states (Bronson, 1985). A growing body of data indicates that annual cyclicity in male testosterone occurs in some baleen whale species (Vu *et al.*, 2015; Hunt *et al.*, 2018, 2022; Cates *et al.*, 2019; Melica *et al.*, 2021), including some resident non-migratory populations (e.g. fin whales, *Balaenoptera physalus*, Carone *et al.*, 2019). However, for most mysticete populations such patterns remain poorly understood (Rolland *et al.*, 2005; Corkeron *et al.*, 2017; Hunt *et al.*, 2019), which limits management of spatially and temporally variable anthropogenic activities that may impact reproductive effort in these populations, such as whale watching, vessel traffic and elevated ocean noise (Stewart *et al.*, 2021, 2022; Pirotta *et al.*, 2023; Hague and McWhinnie, 2024).

Gray whales (*Eschrichtius robustus*) range along the eastern and western coasts of the North Pacific Ocean, with two recognized populations: the Western North Pacific (WNP) and Eastern North Pacific (ENP). These whales migrate annually from high-latitude waters where food is abundant in the summer, to lower latitude overwintering areas that are less productive (Rice and Wolman, 1971; Swartz, 2018). The reproductive cycle of gray whales is closely linked to this migration. Pregnant females initiate the southward migration first, followed by females that recently ovulated, then adult males and finally immature whales (Swartz *et al.*, 2023). Non-pregnant females are theorized to ovulate in November and December, with mating likely occurring during this southbound migration (Rice and Wolman, 1971). Some calves are born during the southbound migration of the following year, but most births occur on the winter grounds in late December or early January (Jones, 1984). The northward migration begins in late January, with newly pregnant females leading, followed by adult males and juveniles (Rice and Wolman, 1971), and finally, in April through May, lactating females with their calves. During summer and fall, most gray whales in the ENP return to their feeding grounds in the Chukchi, Beaufort and the northwestern Bering Seas (Pike, 1962; Swartz *et al.*, 2023). However, a relatively small subgroup of whales, known as the 'Pacific Coast Feeding Group' (PCFG), consisting of approximately 212 individuals (Harris *et al.*, 2022), shortens their migration and feeds along the Pacific coast between the southeast Alaska and northern California from May to November (Swartz *et al.*, 2023). The population trajectory of this group appears to be stable (Harris *et al.*, 2022; Barlow *et al.*, 2024).

Information on gray whale reproductive biology is primarily based on scientific whaling efforts off the central California coast between 1959 and 1969, when over 116 adult female gray whales and 166 male gray whales of various demographic units were killed (Rice and Wolman, 1971). These data indicate that male and female gray whales attain sexual maturity between 5 and 11 years of age, averaging 8 years for both sexes (Rice and Wolman, 1971; Bradford *et al.*, 2010). Females were estimated to gestate for ~13 months, wean calves 6–7 months postpartum (Rice and Wolman, 1971), and typically produce a single calf every two years. Recent advancements in non-lethal collection and analysis of non-plasma biological samples (e.g. blubber, Melica *et al.*, 2021; and faecal samples, Lemos *et al.*, 2020; Fernandez Ajó *et al.*, 2023) have enabled a greater understanding of reproductive profiles, seasonality and variability of reproductive hormones in gray whales. However, much of this research has focused on progesterone (i.e. for pregnancy diagnosis) and cortisol (i.e. for examination of impacts of stressors (Lemos *et al.*, 2022a, 2022b; Pirotta *et al.*, 2023), while patterns of testosterone in males remain understudied.

In this study, we investigated the seasonal variability of testosterone concentration in male PCFG gray whales, using a consecutive eight-year dataset (2016–2023) of individual sightings, drone-based photogrammetry and endocrine analysis of faecal samples collected at the PCFG summer foraging ground off the central Oregon coast, USA. By integrating these datasets and employing generalized additive mixed models (GAMMs), we analyze the variability of faecal testosterone in males in relation to total body length (TL), body condition (body area index, BAI) and day of the year (DOY). We tested the following hypotheses: testosterone concentration varies in relation to (i) TL, which serves as a proxy for age and maturity, (ii) body condition, which reflects nutritional needs to support the energetic demands of reproduction, (iii) DOY, which corresponds to the phenology of the reproductive cycle in gray whales, and (iv) year that may reflect population level changes such as overall prey abundance or broad disturbance.

## Materials and Methods

### Sample collection, field methods and study area

Our study was conducted from 2016 to 2023 during the PCFG foraging seasons (late May to mid-October) along the central Oregon coast, USA (off Newport, 44° 38′13″ N, 124°03′08″ W). Using a 5.4 m, rigid-hulled, inflatable boat, we located whales and photographed individuals for identification purposes. When weather conditions allowed, we also performed unoccupied aircraft systems (UAS) flights for photogrammetry analysis (details below). We collected faecal samples opportunistically with two dipnets (300-μm nylon mesh), recording date, time and location of collection. Faecal material was transferred to 500-ml plastic sterile jars and stored on ice until returned to the lab (~3–6 h), followed by long-term storage at −20°C, until the sample was freeze-dried and assayed (see below). Testosterone assays were performed within 12 months of sample collection. Each sample was collected from an individual when no other whale was in near proximity, the whale was travelling alone, or we have confidently identified which individual produced the faecal sample (i.e. no other whale was observed defecating while approaching to collect the faecal sample). Each sample was then linked to a specific whale using photo-identification (see below). When we obtained multiple samples from the same whale on a single day, we either combined them into one jar before analysis to increase sample mass, or when samples from the same individual were analyzed separately, the sample with larger mass was used for statistical analyses. This research was conducted under the NOAA/NMFS permits #16011 and #21678 issued to John Calambokidis. Drone operations were conducted by a Federal Aviation Authority (FAA) certified private pilots with a Part107 licence or under a Certificate of Authorization (2016-WSA-101-COA).

### Photo-identification, age, sex and reproductive maturity

We photo-identified individual whales by comparing photographs taken in the field with identification catalogues of PCFG gray whales held by the Cascadia Research Collective (Olympia, WA, USA) and the Marine Mammal Institute at Oregon State University. Sex of each whale is determined by sighting history (e.g. as female if previously observed with a calf), genetic analyses from biopsies (Lang *et al.*, 2014) or genetic analyses of the faecal samples (detailed methods in Lemos *et al.*, 2020). Our dataset consisted of 353 faecal samples including females (*n* = 200 from 44 unique individuals), males (*n* = 121 from 34 unique individuals) and samples from animals of unknown sex (*n* = 29 from 17 unique individuals). In this study, we included the observations from males only. Age in years is calculated from the date of first sighting, providing either a known age for those whales that were first sighted as calves or a minimum age estimate for non-calves. Non-calves were assumed to be at least one year old at the time they were identified. Based on the mean age of sexual maturity for the species (Rice and Wolman, 1971), we classified individuals with a known age or minimum age ≥ 8 years as mature, while individuals with a known age < 8 were considered juveniles. For individuals with a minimum age less than 8 years, we determined sexual maturity using their TL, which was derived from the individual growth model described in Pirotta *et al.* (2024) that accounts for photogrammetric uncertainty. Since PCFG reach shorter asymptotic lengths than ENP whales (Bierlich *et al.*, 2023), we estimated the length at maturity of PCFG males using the ratio between the length at maturity and the asymptotic length for ENP males (see Supplementary Material). Individuals were considered mature if at least 50% of the posterior predictive distribution of their TL from Pirotta *et al.* (2024) was greater than the calculated length at maturity for male PCFG gray whales (i.e. >10.69 m).

### Faecal hormone extractions and assays

Faecal samples contain metabolized products of parent testosterone hormone, i.e. faecal androgen metabolites (hereafter: Tm) (Palme, 2005). Tm concentrations from 2016 to 2018 are from Lemos *et al.* (2020), while Tm data from 2019 to 2023 samples are reported for the first time here. Identical methods were used throughout 2016–2023. In brief, faecal samples were filtered, desalinated and freeze-dried, followed by weighing of 0.02–0.05 g of dried, homogenized samples to the nearest 0.001 g (Lemos *et al.*, 2020). Samples <0.02 g were excluded to avoid spurious inflated values associated with the "small sample effect" (Hunt *et al.*, 2006; Fernández Ajó *et al.*, 2022). We extracted hormones from aliquoted faecal samples using 90% methanol (HPLC grade, Fisher Chemical™) and quantified Tm using a commercial testosterone immunoassay kit (Enzo Life Sciences #ADI-900-065) that has been validated specifically for Tm of gray whale faecal samples (Lemos *et al.*, 2020), following the manufacturer's protocols (https://www.enzolifesciences.com). For quality assurance and control, all samples and standards were run in duplicate. Samples were re-analyzed if the optical density between duplicates exceeded a coefficient of variation (CV) of 15%. If a sample’s concentration fell outside the 15–98% percent-bound range, we adjusted the dilution accordingly prior to reanalysis. One sample was below the limit of detection of the assay (<LOD) and was assigned a concentration of half the LOD reported by the manufacturer. The inclusion or exclusion of this sample did not affect the overall results. Final data are expressed as ng of immunoreactive hormone per g of dried faeces.

### UAS-based photogrammetry

We collected aerial videos from four different types of UAS as described in Lemos *et al.* (2022a); the UAS and camera specifications are detailed in Supplementary Material, Table S1. Videos were recorded at an altitude between 20 and 60 m (with one >70 m). No behavioural responses from the whales to the UAS were observed, i.e. no alterations in behaviour such as changes in travel direction or interruption of foraging activities. Individual snapshots of whales were extracted from videos using VLC Media Player (Version 3.16 VideoLAN) and then imported into MorphoMetriX (v1, v2; Torres and Bierlich, 2020) to measure TL and perpendicular body widths in 5% increments of TL. All measurements were processed using CollatriX (Bird and Bierlich, 2020). The length and body widths between 20 and 70% of TL were used to calculate BAI, a length-standardized metric of body condition (Burnett *et al.*, 2019) with low uncertainty allowing for precise comparison of body condition across time and demographic units (Bierlich *et al.*, 2021a). We accounted for photogrammetric uncertainty associated with each UAS in TL and BAI measurements following the methodology outlined by Pirotta *et al.* (2024) and Bierlich *et al.* (2021a, 2021b). To explore the relationship between BAI, TL and variation in faecal testosterone concentrations of individual whales, we assessed BAI measurements alongside faecal Tm data collected from the same whale on the same day, or when UAS photogrammetry images from a whale were not available from the same day as faecal sample collection, we incorporated BAI values measured ±14 days of faecal sample collection from the same individual, assuming that gray whales are not expected to substantially change their body condition size within a two-week period (Soledade Lemos *et al.*, 2020). TL was estimated at the yearly scale (see Pirotta *et al.*, 2024).

### Data analysis

We included only males with complete observations, i.e. morphometrics (BAI and TL) and hormone quantifications (Tm) in our data analyses. Twelve samples were excluded from analysis for the following reasons: five samples were from unknown whales; five samples were from whales of unknown or unconfirmed sex; one sample had insufficient mass for the extraction (< 0.02 g); one sample had an abnormal appearance (mucus consistency with bloody appearance). Faecal Tm concentrations were log-transformed for analyses due to their non-normal distribution. We estimated Tm baselines for each age category (JM and MM) using an iterative process that excludes all data points greater than the mean ± two standard deviations (Mean ± 2SD) until no observations exceed this value, following methods from (Brown *et al.*, 1988). To detect outliers in testosterone levels, we calculated the interquartile range (IQR) by subtracting the first quartile (Q1) from the third quartile (Q3) of the testosterone data. We defined the outlier thresholds as 1.5 times the IQR below Q1 and above Q3. Any values falling below the lower threshold or above the upper threshold were considered outliers. To explore the differences in Tm concentrations and BAI between JM and MM while also controlling for phenological patterns, we segmented the data into three periods of equal duration, each 47 days long during our field seasons between the date of earliest faecal Tm sample collection (May 22) and the latest (October 11): early-season (May 22—July 08), mid-season (July 09—August 25) and late-season (August 26—October 11). As part of exploratory data analysis, we investigated the correlation between TL and age using the Pearson correlation coefficient to determine whether TL could serve as a proxy for age in this study. Additionally, to explore and visualize the relationship between Tm and the potential explanatory variables (TL, age, BAI and DOY), we plotted Tm against each variable and faceted the plots by year (Supplementary Material, Figure S1).

We fitted GAMMs to examine the variability in Tm concentrations as a function of TL, BAI and DOY. GAMMs allow for non-linear relationships between the response and the explanatory variables. Prior to fitting the model, to ensure comparability and robustness in our analyses, we standardized all continuous variables. This involved rescaling each variable to have a mean of 0 and a standard deviation of 1, preventing differences in measurement units from influencing the results. GAMMs were fitted to the log-transformed Tm data, using a Gaussian distribution with an identity link and a restricted maximum likelihood method (REML), in the ‘mgcv’ package for R (R Core Team, 2023; version 1.8–40). Separate thin-plate regression splines with shrinkage were fitted for each demographic unit (JM and MM). Individual whale ID and year of sample collection were included as random effects to account for both individual and annual variability. The tested models were checked using residual diagnostic plots. Model selection involved comparing models with and without random effects using the Akaike Information Criterion (AIC). Once the decision was made regarding random effects, we refitted the models using Maximum Likelihood (ML) estimation. We conducted model selection for fixed effects based on parsimony and goodness of fit using AIC, and finally refitted the selected model using REML for inference.

## Results

### Exploratory data analyses

**Figure 1:**
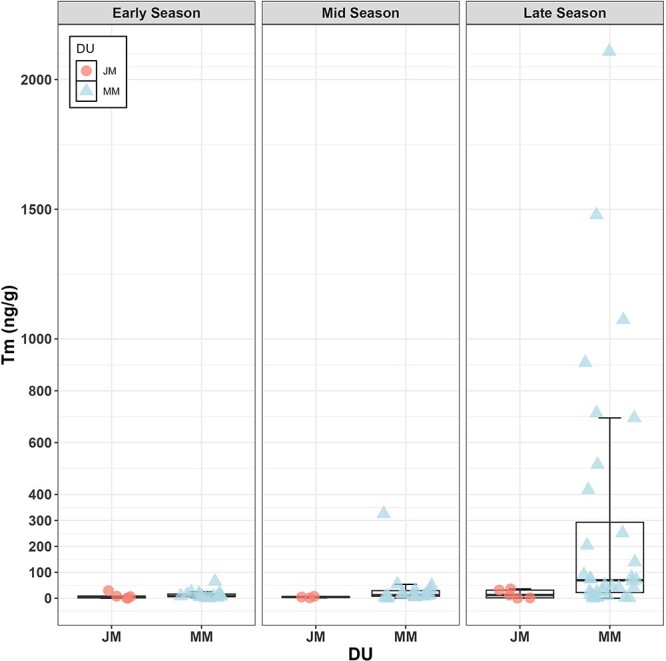
Group mean comparisons for faecal androgen metabolite concentrations (Tm, ng of immunoreactive hormone per g dried faeces) between JM and mature male (MM) gray whales between three periods of the sampling season: early- (May 21 to July 21), mid- (July 23 to August 29) and late-season (August 31 to October 10th). The black horizontal lines represent the group median; the boxes enclose 50% of the data; whiskers enclose the smallest and largest values within 1.5 times the interquartile range below and above the 25th and 75th percentiles, respectively; individual values are shown as circles.

**Figure 2:**
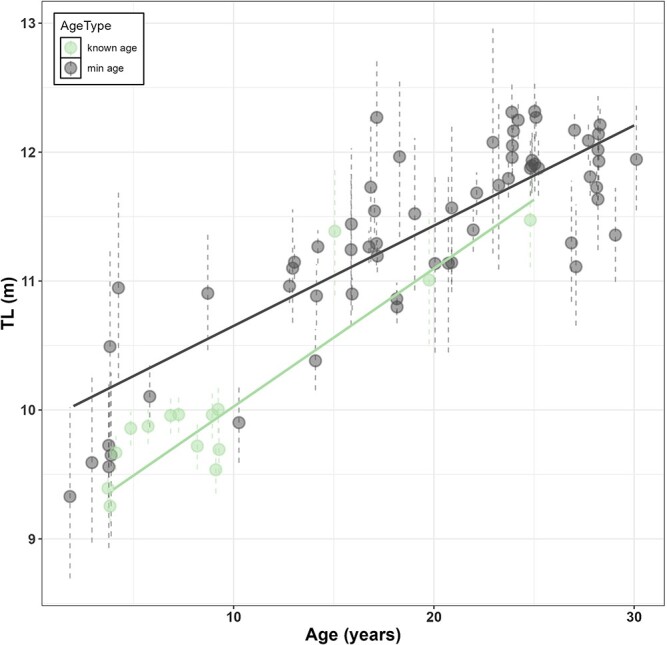
Correlation between age (years) and total length (TL; meters) of gray whales. TL is derived from individual growth curves described in Pirotta *et al.* (2024), where uncertainty is represented by vertical dashed lines showing the posterior 95% credible intervals. Age type is calculated from the date of first sighting, providing either a minimum age estimate (min age) or a known age for whales first sighted as calves. Dark grey closed circles represent observations for whales with a minimum age (*n* = 65), with the grey line showing the linear regression fit. Light green closed circles represent observations for whales with a known age (*n* = 13), with the green line showing the linear regression fit. Both lines were generated using linear regression (method = 'lm').

Our final data set, including only complete observations from males, consisted of 78 faecal Tm quantifications paired with morphometric data (BAI) on the same day or within ±14 days of faecal sample, and a TL measurement for the same year of the sample collection which included 25 individual males, comprising 13 observations from JM and 65 from MM; see Supplementary Material, Table S2. Nine individuals were sampled only once over the eight-year period, but several were sampled twice or more in different years or within a single season (Supplementary Material, Table S3). Notably, one individual was sampled 12 times across the study period (Supplementary Material, Table S3). The mean, median, SD and range (maximum and minimum) for each variable and by demographic group are reported in Table 1. Tm concentrations of MM were highly variable, ranging from a minimum of 0.01 ng/g to a maximum of 2107.77 ng/g (Table 1, Fig. 1). We identified 12 outliers in testosterone levels that exceeded 1.5 times the interquartile range above the third quartile. All these whales were MM, and the samples were collected during the third period of the season (Table 3). Overall and demographic group baseline levels of Tm are shown in Table 2. The BAI range (maximum and minimum) for the three season’s periods for Jm and MM is reported in Table 4. The exploratory plots showing the relationship between Tm and the explanatory variables are provided in Supplementary Material, Figure S1. A strong positive correlation is observed between TL and age (*r* = 0.90, *t*_(76)_ = 16.08, *P* < 0.001; Fig. 2). Based on these results, further analyses included TL as a proxy of age.

**Table 1:** Summary statistics for morphometric variables (TL = total body length, BAI = body area index) and faecal androgen metabolite (Tm) concentrations of gray whales, grouped by demographic unit (JM = juveniles males or MM = mature males)

**JM**						
**Variable**	**Count**	**Mean**	**Median**	**SD**	**Min**	**Max**
**TL (m)**	21	9.62	9.69	0.27	9.10	10.16
**BAI**	13	25.36	25.18	2.78	21.80	29.57
**Tm (ng/g)**	24	11.62	6.85	13.31	0.02	50.89
**MM**						
**TL (m)**	77	11.33	11.34	0.70	9.65	12.11
**BAI**	65	26.69	27.06	2.86	19.09	32.39
**Tm (ng/g)**	86	136.21	19.38	334.51	0.01	2107.77

**Table 2:** Baseline concentration of faecal androgen metabolites (Tm) of gray whales

**Dataset**	**Baseline (ng/g)**	**SD (ng/g)**
**All**	6.48	4.27
**JM**	5.51	4.06
**MM**	6.89	4.43

Top line shows the full dataset (all males); the other lines show data restricted to demographic units (JM = juveniles males or MM = mature males). The Tm baselines are estimated via an iterative process that excluded all data observations greater than the mean ± 2 SD until no points exceeded this maximum value (following Brown *et al.*, 1988).

**Figure 3:**
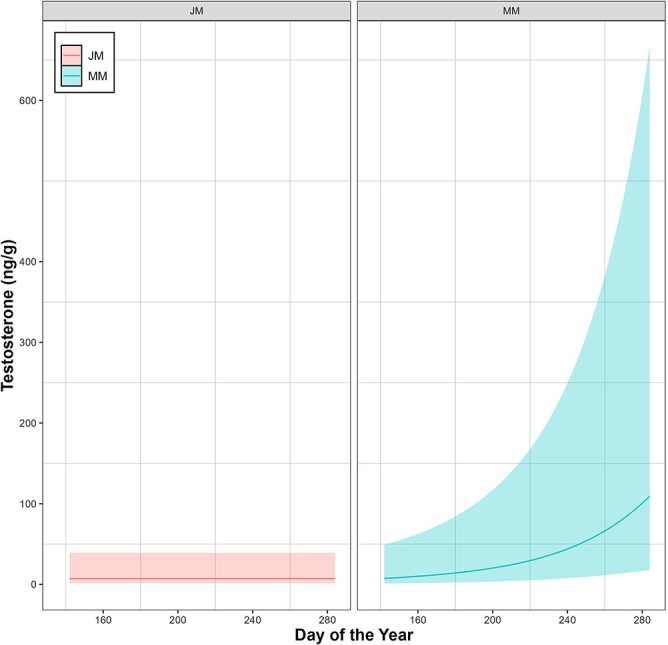
Estimated relationships between faecal testosterone and DOY by demographic unit (juvenile males, JM, on the left; mature males, MM, on the right). Given the inclusion of an individual-level random effect in the final GAM, testosterone concentrations were predicted using the random intercept from one of the males in the sample, which was selected because it had an intercept close to the mean across individuals. The shaded area represents approximate 95% confidence intervals. DOY 160 = May 27 and DOY 280 = October 11.

**Table 3:** Samples identified with testosterone values exceeding 1.5 times the interquartile range of the third quartile

**ID**	**Year**	**DOY**	**DU**	**Age**	**BAI**	**TL**	**Tm**
**Er-0007**	2019	284	MM	25^*^	26.85	11.25	2107.77
**Er-0012**	2018	249	MM	14	32.41	10.62	1478.04
**Er-0369**	2023	252	MM	25	29.12	12.15	1073.63
**Er-0049**	2022	269	MM	13	29.53	11.30	908.09
**Er-0017**	2023	280	MM	18	28.07	11.03	713.77
**Er-0008**	2017	267	MM	17	25.61	11.45	695.37
**Er-0056**	2023	252	MM	27	29.03	11.03	515.54
**Er-0034**	2016	242	MM	16	30.56	11.53	417.96
**Er-0012**	2018	220	MM	14	19.59	10.62	325.75
**Er-0256**	2019	280	MM	25	25.83	11.99	251.25
**Er-0017**	2021	247	MM	16	24.98	11.01	204.25
**Er-0017**	2022	242	MM	17	25.17	11.02	139.57

The table includes ID, which corresponds to each whale’s unique photo-identification catalogue code held by the Marine Mammal Institute at Oregon State University; Year, representing the year of sample collection; DOY, the DOY of sample collection; DU, the demographic unit according to sexual maturity (MM = mature males), Age, as length of sighting history in years, with the asterix indicating an individual of known age (first observed as a calf); BAI, the body area index; TL, the total body length expressed in meters; and Tm, the apparent concentration of testosterone in faecal samples, expressed in ng/g of dry sample.

**Table 4:** BAI range for sexual maturity; MM = mature males and JM = juvenile males by season period; early season- (May 21 to July 21), mid- (July 23 to August 29) and late-season (August 31 to October 10th)

**Period**	**JM**		**MM**	
	**Min**	**Max**	**Min**	**Max**
**Early season**	21.83	29.45	22.55	30.41
**Mid season**	22.38	25.90	19.08	30.73
**Late season**	23.61	29.57	24.27	32.39

### GAMMs

Model selection indicated that individual ID should be retained as a random effect in the model, but not year. For the fixed effects selection, all models were within ΔAIC < 2 and thus can be considered indistinguishable from one another. Therefore, we identified the best model based on the parsimony principle. The most parsimonious model from which to draw inference included only the individual-level random effect and the interaction between DOY and demographic unit (Table 5). Tm concentrations increased with DOY in MM, but not in JM (Table 5, Fig. 1). This model explained 64% of the deviance, with an adjusted *R*-squared value of 0.54 (Fig. 3, and Supplementary Material, “Best **GAMM**”).

**Table 5:** Performance metrics of GAMMs of faecal androgen metabolites (Tm) of gray whales in relation to demographic unit (DU, i.e. juvenile males vs. mature males), body condition (BAI), TL and DOY

**Model REML**	**Response**	**Random effect**	**Predictor**	**dev.exp**	**AIC**	**δAIC**
REML.1	Tm ~	ID + Year	DU + s(BAI) + s(TL) + s(DOY), by = DU	64.3	275.36	3.46
REML.2	Tm ~	ID	DU + s(BAI) + s(TL) + s(DOY), by = DU	64.3	271.90	0.00
**Model ML**	**Response**	**Random effect**	**Predictor**	**dev.exp**	**AIC**	**δAIC**
ML.2	Tm ~	ID	DU + s(BAI) + s(TL) + s(DOY), by = DU	64.3	272.24	0.00
ML.3	Tm ~	ID	DU + s(TL) + s(DOY), by = DU	62.6	272.38	0.14
ML.4	Tm ~	ID	DU + s(DOY), by = DU	62.6	272.38	0.14

Models fitted with REML for the random effects selection are shown on top, and the models fitted with ML for the fixed effects selection on the bottom. Performance metrics include Akaike Information Criterion (AIC) and percent deviance explained (dev.exp). The preferred model (simplest model within 2 units of the lowest AIC) is highlighted (ML.4).

## Discussion

Our study reveals a significant association between the DOY and increased faecal testosterone levels in MM gray whales as the foraging season progresses, whereas no such pattern was observed in juveniles. The mean concentration of faecal testosterone in male gray whales is low in early season and mid-season (May 22nd through August 25th) in both JM and MM but is elevated only in MM in the later season, although not for every MM individual. The finding that Tm remains constant in JM but elevates in MM in the late season confirms that development of male sexual characteristics is age-dependent (Table 1 and Fig. 1). Previous studies based on different sample types (i.e. blubber) and histological examination from whaling data are consistent with our findings. For example, Melica *et al.* (2021) analyzed hormones from blubber biopsies and found that testosterone levels in blubber were elevated in adult male gray whales during the fall season compared to the summer months. Rice and Wolman (1971) examined testes from both immature and adult male gray whales collected during scientific whaling efforts, observing spermatogenesis in the seminiferous tubules of adult males. They also noted wider diameters of the seminiferous tubules and increased testes weights in adult gray whales during the southbound migration. These histological and physiological changes, regulated by increased testosterone levels, suggest that the whales are preparing for breeding during the late foraging season, which may mark the onset of the mating season.

Earlier in the summer (i.e. farther from onset of the breeding season), MM presented low testosterone levels, which are comparable to those of JM whales (Fig. 1). Low androgens in MM in non-breeding seasons have also been documented from analyses of hormone concentrations in baleen of multiple species (i.e. bowhead whale, *Balaena mysticetus*, North Atlantic right whale, *Eubalaena glacialis* and blue whale, *Balaenoptera musculus*; Hunt *et al.*, 2018, 2022). Notably, some males presented extreme high values (Table 3), and all these extreme outliers corresponded to MM in the late season. Though the expected variation in whale testosterone levels remains unclear, we believe that these values could actually be within the normal ranges for actively reproducing MM. Based on these results, we concluded that efforts to determine the age class composition (JM vs. MM) in PCFG gray whales, and likely other seasonally reproducing mammals, based on values of testosterone is only advisable in those months of the year when adult males are expected to have elevated levels of testosterone (e.g. after August 25 in PCFG gray whales). Furthermore, additional data, particularly involving repeated sampling of males of known age as they transition from juvenile to sexually mature, would likely provide more insight into these questions.

While we hypothesized that Tm would increase with age, and therefore with TL, the GAMM analyses did not find TL to be a significant predictor of Tm. Age and TL do exhibit a high positive correlation (Fig. 2), indicating that TL can serve as a reliable proxy for age in male PCFG gray whales. However, in gray whales the age-TL relationship tends to plateau at an asymptotic length (approximately 11.88 m for PCFG males, Bierlich *et al.*, 2023), potentially obscuring the relationship between testosterone levels and age, particularly for older whales. Additionally, recent research indicates that PCFG gray whales are now shorter than they were historically (Pirotta *et al.*, 2024), which might further complicate the detection of trends in the relationship between age and testosterone levels in this study. Future studies might benefit from exploring the relationship between age and Tm with additional methods of determining age, e.g. epigenetic age determination from skin biopsies (Barratclough *et al.*, 2024), or, for stranded specimens, racemization of the eye lens (Hunt *et al.*, 2022).

Although body size and nutritional condition (i.e. BAI) are known to influence testosterone levels (Tm) and reproductive attempts in male mammals (Buck and Barnes, 1999; Hau *et al.*, 2017; Williams *et al.*, 2017), our analysis did not indicate any relationship between BAI and Tm concentrations. However, we cannot rule out the possibility that the lack of correlation is an artefact of our limited sampling range of BAI during the late season. The minimum BAI value included in this analysis was 19.09 for a whale sampled in mid-season, while most early-season whales have a BAI ranging from 21.8 to 30.4 (Table 4). These early-season whales are expected to be returning from the wintering grounds, where food is scarce, and they are expected to be nutritionally limited. In our study, all the MM whales in the late season had a BAI greater than 29.57, close to the upper limit of the BAI range for whales in the early season, which might indicate that no MM in our study were in poor body condition towards the end of the sampling season. However, not all MM presented elevated Tm in the late season, raising questions about which other factors might influence reproductive attempts in male PCFG whales.

Identifying the onset of the reproductive season in male whales through testosterone levels assessment can provide insights into the timing and location of the conceptive season, which is relevant for management efforts. Our study found a positive correlation between DOY and Tm. The observed rise of Tm in the MM over time likely indicates preparation for mating while still at the foraging grounds, as the southbound migration nears. Notably, some MM showed a sharp increase in Tm levels in the late season, after approximately DOY 217 (~ August 5th; Supplementary Material, Figure S.3 and Table 3). These findings underscore the importance of the PCFG range not only as a foraging area but also a significant site for male reproductive preparation, particularly in the late season.

Moreover, the findings presented here, along with existing data on the migration phenology and genetic population structure of gray whales, suggest that the use of different foraging grounds across the North Pacific during the summer may influence reproductive segregation between populations. For instance, satellite tagging data indicates that whales migrating from the WNP to the wintering lagoons in Baja California, Mexico, remain far to the west during the theorized peak conception period in late November to early December (Rice and Wolman, 1971; Mate *et al.*, 2015), making it unlikely for them to mate with ENP or PCFG whales. Genetic studies have revealed significant mitochondrial and nuclear genetic differentiation between WNP and ENP gray whales, implying minimal interbreeding and suggesting assortative mating based on location and migratory timing (Lang *et al.*, 2022). In contrast, no significant nuclear DNA differences were found between PCFG and other ENP whales, indicating at least some degree of reproductive mixing between these groups (Lang *et al.*, 2014; D’Intino *et al.*, 2023). However, although the exact migration patterns of PCFG whales remain unknown, evidence of associations among individual PCFG whales during their southbound migration has been observed (Calambokidis and Perez, 2017). Thus, our findings, which indicate that MM are reproductively prepared while in PCFG foraging grounds late in the season, and consequently during their southbound migration when they interact with mature females, add to the evidence that the PCFG may be somewhat reproductively isolated from ENP whales. While current data cannot conclusively prove this reproductive segregation, further research is needed to explore this hypothesis, given its important management and conservation implications.

In conclusion, our findings reveal a significant association between the DOY and increased Tm levels in MM, while no such pattern was observed in JM. These results offer insights into the timing for the onset of the reproductive season for PCFG gray whales, highlight the foraging grounds of central Oregon as potentially important areas for males preparing for reproduction, particularly towards the end of the summer, and demonstrate the value of non-invasive faecal sampling to enhance the understanding of population dynamics within the PCFG gray whale subgroup.

## Supplementary Material

ESM_Fernandez_Ajo_et_al_2024_final_coae094

## Data Availability

The data underlying this article are available in fig**share** digital repository, at https://figshare.com/s/acac25d8ab46cf6b7073.
