# Supplementary material for: Variation in faecal testosterone levels in male gray whales on a foraging ground relative to maturity and timing
Source: Conserv Physiol. 2025 Jan 20;13(1):coae094. doi: 10.1093/conphys/coae094 (PMC11744369; doi:10.1093/conphys/coae094)
Supplement: ESM_Fernandez_Ajo_et_al_2024_final_coae094 [file esm_fernandez_ajo_et_al_2024_final_coae094.pdf]

Supplementary material for manuscript: "Variation in fecal testosterone levels in male gray whales on a foraging ground relative to maturity and timing."

Authors: Fernandez Ajó, A.[1], Buck C.L.[2], Hunt K.E.[3], Pirotta E.[4], New L.[5], Dillon D.[2,a], Bierlich, K.C.[1], Hildebrand, L[1], Bird, C.N.[1], Torres, L.G.[1]

[1]Geospatial Ecology of Marine Megafauna Lab, Marine Mammal Institute, Department of Fisheries, Wildlife and Conservation Sciences, Oregon State University, Newport (97365), Oregon, USA;
[2]Department of Biological Sciences, Northern Arizona University, 617 S. Beaver St., Flagstaff, AZ 86011, USA;
[3]Smithsonian-Mason School of Conservation & Department of Biology, George Mason University, 1500 Remount Rd, Front Royal, VA 22630, USA
[4]Centre for Research into Ecological and Environmental Modelling, Univ. of St Andrews, St Andrews, UK;
[5]Ursinus College, 601 E Main St, Collegeville, PA 19426, USA.
[a]Present address: New England Aquarium, 1 Central Wharf, Boston, MA 02110.

Calculation of the length at maturity for male PCFG

For PCFG whales in our dataset with a minimum age estimate less than 8 years, we used total body length to classify maturity. Rice & Wolman, (1971) determined that the average length at maturity for male ENP gray whales is 11.1 m. A recent comparison of length-at-age growth curves between ENP and PCFG gray whales found that PCFG whales reach shorter asymptotic length than ENP whales (Bierlich et al., 2023). Specifically, ENP males reach a mean asymptotic length of 12.34 m, whereas PCFG males only reach 11.88 m. To account for potential size differences at maturity between these two groups, we used the ratio of length at maturity to the asymptotic length of ENP males to determine the length at maturity for PCFG males. We first calculate this ratio, *R*, for ENP whales:

$$R_{ENP} = \frac{L_{m,ENP}}{L_{a,ENP}}$$

where L_m is the length at maturity and L_a is the asymptotic length for ENP males. We then assume the ratio for ENP whales is equal to the ratio for PCFG whales

$$R_{ENP} = R_{PCFG}$$

and then use the R_{ENP} to calculate the PCFG length at maturity, L_m,

$$L_{m,PCFG} = L_{a,PCFG} \times R_{ENP}$$

where L_a is the asymptotic length for PCFG males. Following these equations and values provided above, we estimate PCFG males reach maturity at a total body length of 10.69 m.

Table S1. Camera specifications associated with each unoccupied aircraft system (UAS) used in this study. Each UAS had a barometer to record the altitude of the drone during video collection, while the Inspire 2 also had a laser altimeter (LiDAR, LidarBoX, (Bierlich et al., 2023; Dawson et al., 2017)).

UAS	Year	Sensor (mm)	Pixel resolution (px)	Focal length lens (mm)	Altimeter
Phantom 3 Pro	2016-2017	6.16 x 4.6	3840 x 2160	3.61	Barometer
Phantom 4	2016-2017	6.16 x 4.6	3840 x 2160	3.61	Barometer
Phantom 4 Pro	2017-2019	13.2 x 8.8	3840 x 2160	8.8	Barometer
Inspire 2	2020-2023	17.3 x 13	3840 x 2160	25	Barometer & LiDAR/LidarBoX

Table S2. Complete observations for male PCFG gray whale collected between 2016-2023. Information includes ID = corresponds to each whale's unique photo-identification catalogue code held by the Marine Mammal Institute at Oregon State, Year = year of sample collection, DOY = day of the year for the sample collection, Period = periods of the sampling season: early- (May 21 to July 21), mid- (July 23 to August 29), and late-season (August 31 to October 10th). Age = length of sighting history, Age Type = calculated from the date of first sighting, providing either a minimum age estimate (min age) or a known age for those whales that were first sighted as calves (known age), DU = demographic unit according to the sexual maturity; MM = mature males and JM = juvenile males. BAI = body area index. TL = total body length expressed in meters. Tm = apparent concentration of testosterone in fecal samples expressed in ng/g of dry sample.

ID	year	DOY	Period	Age	AgeType	DU	BAI	TL	Tm
Er-0006	2016	241	Late Season	21	min age	MM	30.45	11.77	80.53
Er-0022	2016	242	Late Season	17	min age	MM	26.00	12.07	26.97
Er-0033	2016	242	Late Season	18	min age	MM	30.43	11.75	0.08
Er-0034	2016	240	Late Season	16	min age	MM	28.85	11.53	6.66
Er-0034	2016	242	Late Season	16	min age	MM	30.56	11.53	417.96
Er-0208	2016	239	Late Season	4	min age	JM	29.53	9.60	12.49
Er-0223	2017	182	Early Season	2	min age	JM	21.81	9.49	0.02
Er-0342	2017	194	Mid Season	17	min age	MM	19.10	11.37	0.01
Er-0342	2017	200	Mid Season	17	min age	MM	19.10	11.37	4.35
Er-0008	2017	267	Late Season	17	min age	MM	25.61	11.45	695.37
Er-0036	2017	267	Late Season	15	known age	MM	28.26	11.22	73.53
Er-0017	2018	152	Early Season	13	min age	MM	22.55	10.97	1.97
Er-0223	2018	171	Early Season	3	min age	JM	25.43	9.69	29.35
Er-0256	2018	163	Early Season	24	min age	MM	25.03	11.99	2.74
Er-0256	2018	151	Early Season	24	min age	MM	25.03	11.99	7.80
Er-0295	2018	173	Early Season	4	known age	JM	29.43	9.11	1.44
Er-0348	2018	163	Early Season	28	min age	MM	27.15	11.81	7.80
Er-0353	2018	163	Early Season	4	min age	MM	27.73	10.79	1.28
Er-0012	2018	220	Mid Season	14	min age	MM	19.59	10.62	325.75
Er-0353	2018	213	Mid Season	4	min age	MM	27.84	10.79	7.80
Er-0006	2018	250	Late Season	23	min age	MM	27.01	11.78	67.11
Er-0012	2018	249	Late Season	14	min age	MM	32.41	10.62	1,478.04
Er-0015	2018	263	Late Season	20	min age	MM	27.10	11.25	2.70
Er-0017	2018	244	Late Season	13	min age	MM	26.03	10.97	75.33
Er-0049	2018	250	Late Season	9	min age	MM	26.89	11.18	90.96
Er-0256	2018	262	Late Season	24	min age	MM	27.93	11.99	3.41

ID	year	DOY	Period	Age	AgeType	DU	BAI	TL	Tm
Er-0256	2018	271	Late Season	24	min age	MM	27.93	11.99	9.33
Er-0348	2018	263	Late Season	28	min age	MM	28.75	11.81	47.03
Er-0015	2019	183	Early Season	21	min age	MM	25.66	11.26	8.86
Er-0017	2019	189	Early Season	14	min age	MM	27.41	10.99	19.99
Er-0058	2019	203	Mid-Season	23	min age	MM	28.63	11.78	11.95
Er-0007	2019	284	Late Season	25	known age	MM	26.85	11.25	2,107.77
Er-0034	2019	280	Late Season	19	min age	MM	24.29	11.55	39.21
Er-0223	2019	255	Late Season	4	min age	JM	28.36	9.85	31.32
Er-0223	2019	263	Late Season	4	min age	JM	28.36	9.85	36.12
Er-0256	2019	280	Late Season	25	min age	MM	25.83	11.99	251.25
Er-0210	2020	205	Mid-Season	4	known age	JM	22.37	9.38	4.88
Er-0210	2020	216	Mid-Season	4	known age	JM	23.76	9.38	1.47
Er-0348	2020	214	Mid-Season	30	min age	MM	23.87	11.82	4.04
Er-0256	2021	142	Early Season	27	min age	MM	23.68	11.99	4.70
Er-0315	2021	160	Early Season	6	min age	JM	22.07	10.18	6.24
Er-0342	2021	228	Mid-Season	21	min age	MM	25.83	11.39	7.48
Er-0017	2021	247	Late Season	16	min age	MM	24.98	11.01	204.25
Er-0210	2021	246	Late Season	5	known age	JM	23.62	9.58	1.62
Er-0342	2022	153	Early Season	22	min age	MM	23.03	11.40	7.87
Er-0342	2022	166	Early Season	22	min age	MM	23.61	11.40	25.00
Er-0369	2022	153	Early Season	24	min age	MM	25.80	12.15	64.87
Er-0017	2022	234	Mid-Season	17	min age	MM	24.40	11.02	53.63
Er-0036	2022	225	Mid-Season	20	known age	MM	21.55	11.28	21.73
Er-0256	2022	191	Mid-Season	28	min age	MM	22.74	11.99	13.59
Er-0256	2022	196	Mid-Season	28	min age	MM	22.74	11.99	1.49
Er-0256	2022	234	Mid-Season	28	min age	MM	24.86	11.99	12.72
Er-0295	2022	196	Mid-Season	8	known age	MM	25.68	9.68	49.12
Er-0017	2022	242	Late Season	17	min age	MM	25.17	11.02	139.57
Er-0049	2022	269	Late Season	13	min age	MM	29.53	11.30	908.09
Er-0080	2022	247	Late Season	29	min age	MM	29.48	11.10	26.95
Er-0210	2022	266	Late Season	6	known age	JM	25.17	9.74	0.45
Er-0256	2022	264	Late Season	28	min age	MM	27.73	11.99	2.11
Er-0256	2022	266	Late Season	28	min age	MM	27.73	11.99	24.55
Er-0256	2022	277	Late Season	28	min age	MM	26.26	11.99	14.95
Er-0369	2022	244	Late Season	24	min age	MM	27.44	12.15	49.56

ID	year	DOY	Period	Age	AgeType	DU	BAI	TL	Tm
Er-0210	2023	189	Early Season	7	known age	JM	23.87	9.87	7.90
Er-0369	2023	146	Early Season	25	min age	MM	26.94	12.15	14.17
Er-0369	2023	154	Early Season	25	min age	MM	26.94	12.15	12.53
Er-0369	2023	174	Early Season	25	min age	MM	30.42	12.15	18.48
Er-0369	2023	175	Early Season	25	min age	MM	30.42	12.15	8.20
Er-0017	2023	218	Mid-Season	18	min age	MM	29.26	11.03	34.92
Er-0210	2023	190	Mid-Season	7	known age	JM	25.92	9.87	7.46
Er-0214	2023	219	Mid-Season	10	min age	MM	29.85	9.85	10.53
Er-0295	2023	218	Mid-Season	9	known age	MM	30.73	9.76	8.17
Er-0295	2023	234	Mid-Season	9	known age	MM	28.37	9.76	11.76
Er-0369	2023	218	Mid-Season	25	min age	MM	30.28	12.15	31.57
Er-0017	2023	280	Late Season	18	min age	MM	28.07	11.03	713.77
Er-0056	2023	241	Late Season	27	min age	MM	27.06	11.03	71.83
Er-0056	2023	252	Late Season	27	min age	MM	29.03	11.03	515.54
Er-0295	2023	241	Late Season	9	known age	MM	28.37	9.76	7.92
Er-0295	2023	252	Late Season	9	known age	MM	28.37	9.76	41.09
Er-0369	2023	252	Late Season	25	min age	MM	29.12	12.15	1,073.63

Table S3. List of fecal sample events for male PCFG gray whale collected between 2016-2023. Information includes ID = corresponds to each whale's unique photo-identification catalogue code held by the Marine Mammal Institute at Oregon State, and year of the sample collection. Each cell indicates the total count of samples collected for each individual in a given season. The "Total" column indicates the grand total of fecal samples acquired for each individual across the study period (2016-2023).

ID	2016	2017	2018	2019	2020	2021	2022	2023	Total
**Er-0256**	0	0	4	1	0	1	6	0	**12**
**Er-0017**	0	0	2	1	0	1	2	2	**8**
**Er-0369**	0	0	0	0	0	0	2	6	**8**
**Er-0210**	0	0	0	0	2	1	1	2	**6**
**Er-0295**	0	0	1	0	0	0	1	4	**6**
**Er-0342**	0	2	0	0	0	1	2	0	**5**
**Er-0223**	0	1	1	2	0	0	0	0	**4**
**Er-0034**	2	0	0	1	0	0	0	0	**3**
**Er-0348**	0	0	2	0	1	0	0	0	**3**
**Er-0006**	1	0	1	0	0	0	0	0	**2**
**Er-0012**	0	0	2	0	0	0	0	0	**2**
**Er-0015**	0	0	1	1	0	0	0	0	**2**
**Er-0036**	0	1	0	0	0	0	1	0	**2**
**Er-0049**	0	0	1	0	0	0	1	0	**2**
**Er-0056**	0	0	0	0	0	0	0	2	**2**
**Er-0353**	0	0	2	0	0	0	0	0	**2**
**Er-0007**	0	0	0	1	0	0	0	0	**1**
**Er-0008**	0	1	0	0	0	0	0	0	**1**
**Er-0022**	1	0	0	0	0	0	0	0	**1**
**Er-0033**	1	0	0	0	0	0	0	0	**1**
**Er-0058**	0	0	0	1	0	0	0	0	**1**
**Er-0080**	0	0	0	0	0	0	1	0	**1**
**Er-0208**	1	0	0	0	0	0	0	0	**1**
**Er-0214**	0	0	0	0	0	0	0	1	**1**
**Er-0315**	0	0	0	0	0	1	0	0	**1**

Figure S1. Exploratory plots show the relationships between the log-transformed fecal testosterone concentration and the potential predictor variables: total length (top left), age (top right), body area index (BAI, bottom left), and day of the year (DOY, bottom right). Triangle shapes correspond to observations from whales with a minimum age estimate (min age), and closed circles represent individuals of known age. The color indicates the age class, with immature males in salmon (JM) and mature males in blue (MM).

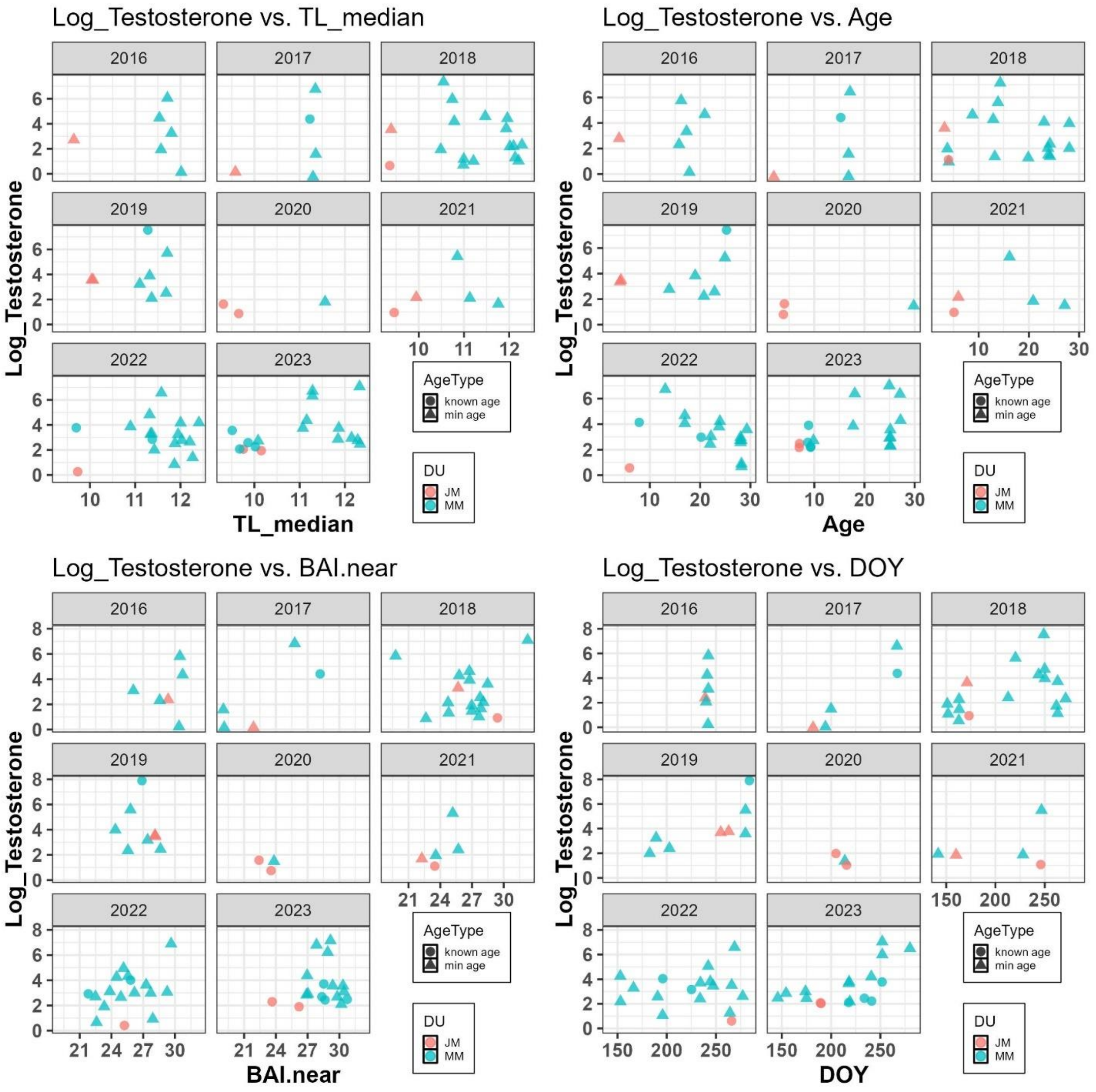

Figure S2. Visualization of best Generalized Additive Mixed Model (GAMM) Fit with REML. The model **explained 63.8% of the deviance**, with an **adjusted R-squared value of 0.54.** The significant effects included the random effect of whale_ID and the interaction between day of the year and demographic unit (DU-MM). The GAMM analysis revealed that the random effect of whale_ID and the interaction between DOY and DU significantly influenced Tm levels in sexually mature gray whales. The other smooth terms did not show significant effects in this model.

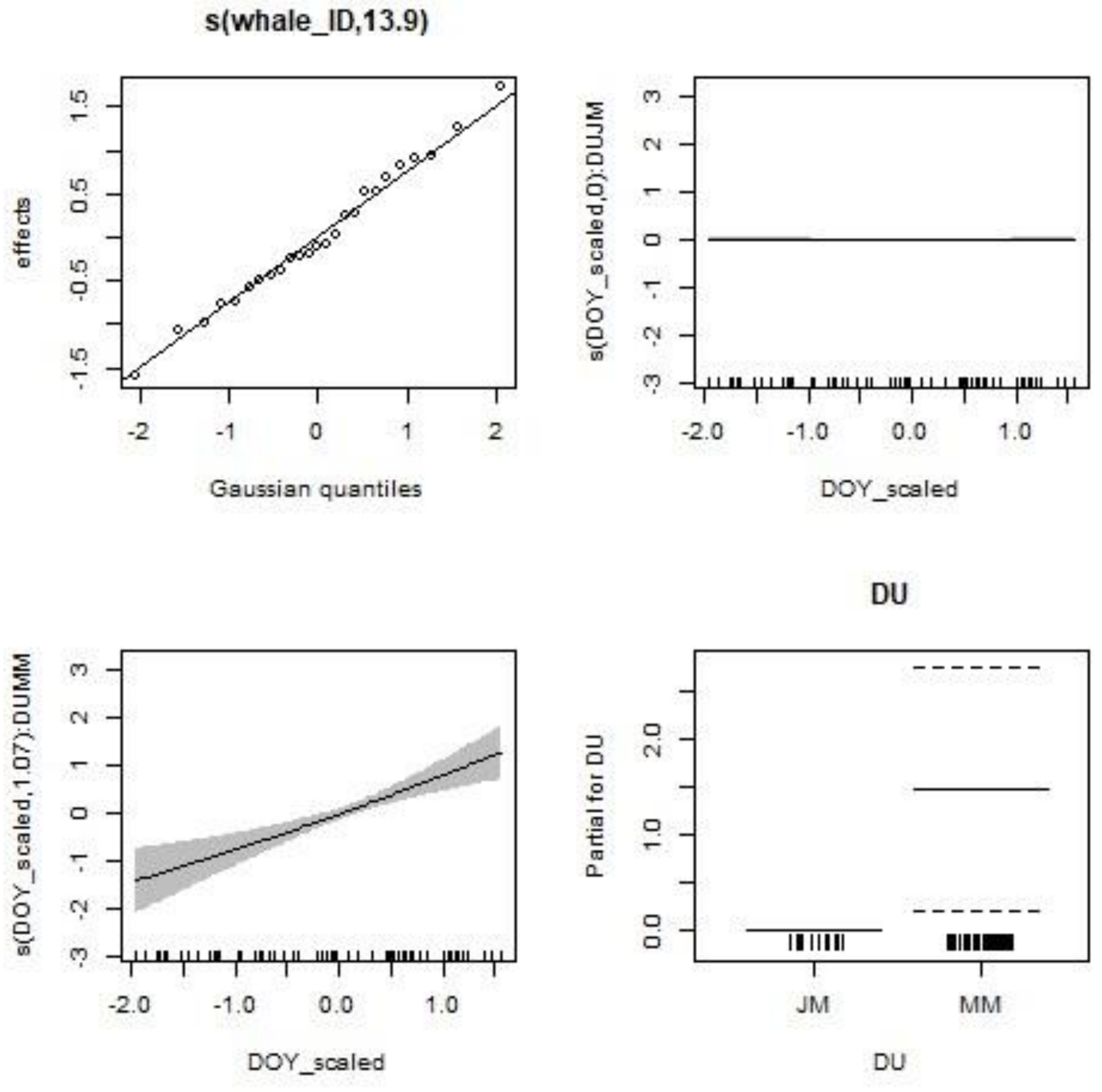

Figure S3. Fecal testosterone concentration (ng*g-1) vs. day of the year (DOY) for mature males (MM, blue triangles) and juvenile males (JM, salmon circles). The vertical dotted blue line indicates a putative date for the onset of the reproductive season for PCFG gray whales DOY = 217. Descriptively, testosterone values in some MM increase sharply after this (i.e., around August 5th).

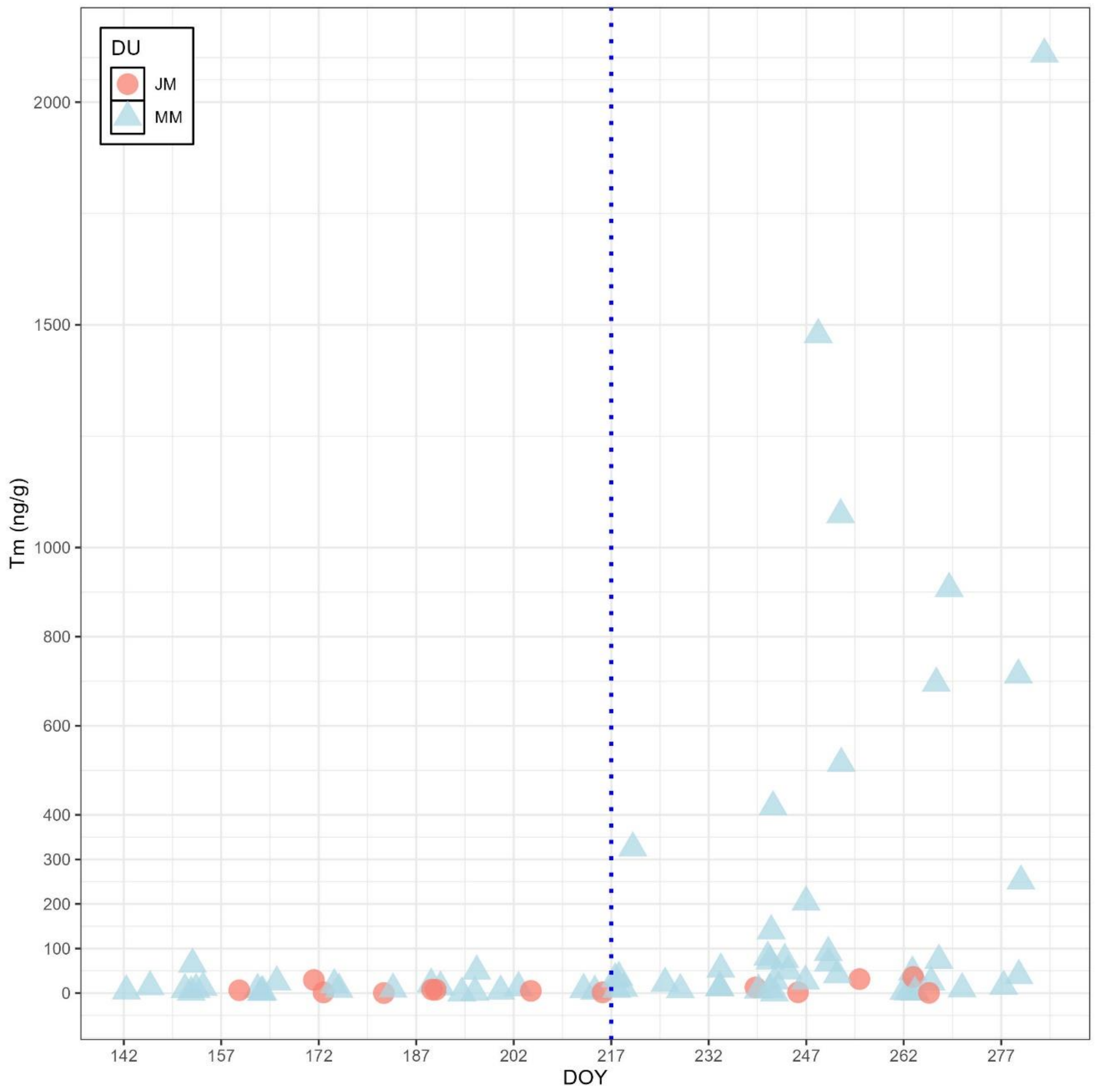

REFERENCES:

Bierlich, K. C., Kane, A., Hildebrand, L., Bird, C. N., Fernandez Ajo, A., Stewart, J. D., Hewitt, J., Hildebrand, I., Sumich, J., & Torres, L. G. (2023). Downsized: gray whales using an alternative foraging ground have smaller morphology. *Biology Letters*, *19*(8). https://doi.org/10.1098/rsbl.2023.0043

Dawson, S. M., Bowman, M. H., Leunissen, E., & Sirguey, P. (2017). Inexpensive Aerial Photogrammetry for Studies of Whales and Large Marine Animals. *Frontiers in Marine Science*, *4*(NOV). https://doi.org/10.3389/fmars.2017.00366

Rice, D. W., & Wolman, A. A. (1971). Life history and ecology of the gray whale (Eschrichtius robustus). In *American Society of Mammalogists*. American Society of Mammalogists.
